# The Carbohydrate-linked Phosphorylcholine of the Parasitic Nematode Product ES-62 Modulates Complement Activation*

**DOI:** 10.1074/jbc.M115.702746

**Published:** 2016-04-04

**Authors:** Umul Kulthum Ahmed, N. Claire Maller, Asif J. Iqbal, Lamyaa Al-Riyami, William Harnett, John G. Raynes

**Affiliations:** From the ‡ Department of Immunology and Infection, Faculty of Infectious and Tropical Diseases, London School of Hygiene and Tropical Medicine, Keppel Street, London WC1E 7HT and; the § Strathclyde Institute of Pharmacy and Biomedical Sciences, University of Strathclyde, Glasgow G4 0RE, United Kingdom

**Keywords:** C1Q complex (C1QA), carbohydrate function, complement, parasite, pentraxin, C-reactive protein, ES-62, acute phase reactant, immunomodulation, phosphorylcholine

## Abstract

Parasitic nematodes manufacture various carbohydrate-linked phosphorylcholine (PCh)-containing molecules, including ES-62, a protein with an *N*-linked glycan terminally substituted with PCh. The PCh component is biologically important because it is required for immunomodulatory effects. We showed that most ES-62 was bound to a single protein, C-reactive protein (CRP), in normal human serum, displaying a calcium-dependent, high-avidity interaction and ability to form large complexes. Unexpectedly, CRP binding to ES-62 failed to efficiently activate complement as far as the C3 convertase stage in comparison with PCh-BSA and PCh-containing *Streptococcus pneumoniae* cell wall polysaccharide. C1q capture assays demonstrated an ES-62-CRP-C1q interaction in serum. The three ligands all activated C1 and generated C4b to similar extents. However, a C2a active site was not generated following ES-62 binding to CRP, demonstrating that C2 cleavage was far less efficient for ES-62-containing complexes. We proposed that failure of C2 cleavage was due to the flexible nature of carbohydrate-bound PCh and that reduced proximity of the C1 complex was the reason that C2 was poorly cleaved. This was confirmed using synthetic analogues that were similar to ES-62 only in respect of having a flexible PCh. Furthermore, ES-62 was shown to deplete early complement components, such as the rate-limiting C4, following CRP interaction and thereby inhibit classical pathway activation. Thus, flexible PCh-glycan represents a novel mechanism for subversion of complement activation. These data illustrate the importance of the rate-limiting C4/C2 stage of complement activation and reveal a new addition to the repertoire of ES-62 immunomodulatory mechanisms with possible therapeutic applications.

## Introduction

Phosphorylcholine (PCh)^4^ is linked to a variety of structures in a range of microorganisms. Filarial nematode products are particularly heavily substituted with PCh and include a number of somatic and excreted antigens of medically important human parasites, such as *Brugia malayi* (and also the feline parasite *Brugia pahangi*) (1, 2), *Onchocerca volvulus* (3), and *Wuchereria bancrofti* (4). Most studied of the PCh-containing molecules is ES-62, a major secreted product of the adult stage of the rodent filarial nematode *Acanthocheilonema viteae*, which acts as a model for human filarial nematode PCh-containing proteins. This tetrameric glycoprotein has PCh attached via *N*-glycan terminal linkage, and each ES-62 molecule can have at least two PCh per glycan (5, 6). ES-62 has a broad immunomodulatory activity, such as the ability to inhibit lymphocyte responses and macrophage activation (7, 8), and PCh is required for this activity (7, 9). The molecule is currently under study for its therapeutic potential because it has been shown to reduce inflammation in a number of models of inflammatory disease; for example, collagen-induced arthritis, which acts as a model for rheumatoid arthritis (10, 11).

We were therefore interested in examining which host proteins would interact immediately with ES-62 following its secretion and appearance in the bloodstream. This is a question that is of relevance to human filariasis because PCh-containing antigens are known to appear in the serum of filarial nematode-infected individuals (12). One candidate was C-reactive protein (CRP) because this protein binds to PCh on bacteria, including *Streptococcus pneumoniae*, which incorporates PCh into its cell wall polysaccharide (CWPS) (13). However, in contrast to the well characterized binding of CRP to bacteria, binding to parasites and parasite ligands is poorly studied. Additionally, it is of interest that the PCh on ES-62 is in a phosphodiester linkage, and such compounds have been reported to have a 10-fold reduced binding capacity for CRP compared with monoesters (14). For *S. pneumoniae*, the binding of CRP is detrimental because it can potentially lead to killing of the bacteria by complement activation (15), and endogenous mouse CRP deficiency can lead to a great increase in susceptibility (16). In contrast however, and as alluded to above, PCh structures present on or secreted by a wide range of nematodes and other parasites can have beneficial roles for the parasites, in particular for evasion of the immune response (17, 18). CRP is known as an important component of the innate immune response and a clinical marker of systemic inflammation because of its dramatic rise following infection, trauma, or other pathology, when it may increase in concentration by at least 1000-fold from a baseline concentration, which is often less than 1 μg/ml. This induction is driven within 24–48 h by a variety of inflammatory cytokines, but interleukin 6 is essential, and interleukin 1 greatly enhances responses (19).

CRP is composed of a pentameric ring of subunits of ∼25 kD arranged in the same plane, with each subunit in the same orientation (20). When CRP binds to PCh or other ligands, it has been shown to activate complement through C1q binding as for the classical pathway. The central pore and cleft on the other side of the pentamer from the ligand-binding site is involved in binding to the globular headgroup of C1q (21, 22). Binding of CRP to factor H and C4 binding protein is not observed at normal concentrations of the native form or ligand-bound CRP (23–26). Therefore, the second aim of this study was to examine the activation of complement by ES-62 because this appeared counterintuitive for the filarial parasite and, in particular, for a molecule that has immunomodulatory and anti-inflammatory activity. Activation of complement and, in particular, C3 cleavage is important not just for innate immunity but also for driving T cell responses (27) and for humoral immunity (28). We demonstrate that, despite strong binding for CRP, ES-62 did not generate a C3 convertase because of the unusual presentation of a CRP ligand at the end of a highly extended and flexible 30-Å glycan structure. This is very different in terms of spatial rigidity to PCh directly coupled to BSA or found in bacterial carbohydrates such as C polysaccharide and thereby constitutes a novel immune evasion strategy. This was confirmed by demonstrating that the same properties are displayed by synthetic analogues that resemble ES-62 only in possessing mobile PCh.

## Experimental Procedures

### Materials

Serum was obtained from normal healthy donors at the London School of Hygiene and Tropical Medicine under informed consent and ethical approval and assayed for CRP by ELISA. Highly purified, endotoxin-free ES-62 was generated from culture supernatants of adult *A. viteae* as described previously (29). Purified CWPS was obtained from the Statens Serum Institute (Copenhagen, Denmark). PCh-BSA was generated as described previously (28). Native CRP and SAP were purified from serum as described previously (30, 31). Mouse myeloma protein, TEPC-15, which recognizes PCh, was obtained from Sigma. Mouse monoclonal antibody 2C10 to native human CRP was provided by Dr. L. Potempa. C1q-deficient serum was supplied by Quidel. Antibody to the active site of C2a (175–62) was provided by Genentech.

### ES-62 and Serum Protein Binding Assays

#### 

##### Pentraxin Binding Assays

Nunc Maxisorb plates were coated with ES-62, PCh-BSA, or CWPS at the stated concentrations in PBS (pH 7.4) overnight at 4 °C. Blocking was performed with 1% w/v BSA in PBS containing 0.05% v/v Tween 20. Purified CRP or human serum diluted as stated in HEPES-buffered saline containing 1 mm CaCl_2_ and 0.05% v/v Tween 20 (HBSCT) was added to wells for 1 h at 20 °C. In some experiments, 10 mm EDTA or 50 mm phosphorylcholine was added. The plate was washed in HBSCT, and bound CRP was detected with polyclonal anti-CRP-HRP conjugate (Dako), followed by washing and 1,1,3,3 tetramethylbenzidine substrate (0.1 mg/ml, Fisher) in 50 mm citrate-phosphate buffer (pH 4.5) containing 0.005% H_2_O_2_) detection at 450 nm. Alternatively, CRP was detected with monoclonal anti-human CRP (2C10) recognizing native CRP. After washing, this was detected with anti-mouse IgG-HRP (Sigma). To analyze serum amyloid P (SAP) binding, we used the same detection protocol but replaced anti-CRP with monoclonal anti-SAP (5.4A, Millipore).

##### Ficolin-2 Binding Assay

Nunc Maxisorb plates were coated with ES-62 or acetylated BSA (Sigma) ligands at the indicated concentrations. These were then each blocked with 10 mm HEPES-buffered saline with 1 mm CaCl_2_ (HBSC) containing 2% (w/v) BSA and washed in HBSCT. Human serum was diluted 1/20 with either HBSCT or HBST (HEPES-buffered saline containing 0.05% (v/v) Tween 20) containing 5 mm EDTA and then incubated on the plate for 1 h at room temperature. Following further washes in the appropriate buffer, biotinylated anti-ficolin 2 (R&D Systems), diluted 1/200, was added to each well, and the plates were incubated for 1 h at room temperature. Binding was detected with streptavidin-peroxidase (BIOSOURCE (1/15,000) and 1,1,3,3 tetramethylbenzidine substrate as described previously.

### Serum Pulldown Assays

100 μl of amine-coated magnetic beads (10 mg/ml, 1-μm diameter, MoBiTec) was incubated with 5 μl of 10 mg/ml fresh 1-ethyl-3-(3-dimethylaminopropyl) carbodiimide in 0.1 m MES buffer (pH 4.5) with 25 μl of 250 μg/ml ES-62 or 1 mg/ml AGP-PCh in the same buffer. Beads were incubated with rolling at room temperature overnight before being washed with PBS and a magnetic activated cell-sorting magnet. Beads were blocked with BSA and incubated with serum, 1 ml from pooled normal donors, for 2 h at room temperature with rolling, washed five times with 10 mm HEPES (pH 7.4) containing 1 mm CaCl_2_, and then eluted with 50 μl of 10 mm EDTA in HEPES buffer or directly into sample buffer. The supernatant and beads were boiled in SDS sample buffer before SDS-PAGE (11%) gel analysis using Coomassie Blue or Western blotting.

### Surface Plasmon Resonance

A Biacore 3000 was used to immobilize 300 response units of ES-62 on a CM5 chip (Biacore, Stevenage, UK). To achieve this, ES-62 (20 μg/ml in 10 mm sodium acetate buffer (pH 5.5)) was coupled via standard amine coupling. Test and control flow cells were blocked with ethanolamine, and the binding experiments were performed in HBS containing 1 mm CaCl_2_ and 0.005% surfactant P20 (pH 7.4). Various concentrations of CRP (10 μg/ml-20 ng/ml) were passed through the flow cells at 10 μl/min for 3 min and allowed to dissociate for a further 5 min.

Binding of ES-62 to immobilized CRP was examined by initially attaching streptavidin (in 10 mm acetate buffer (pH 5.5)) to 5000 response units to flow cells 1–4 of a CM5 chip using amine coupling and ethanolamine deactivation. Biotinylated CRP and SAP were generated by reaction with NHS-LC-biotin (Cayman Biochemicals) in PBS (pH 7.4), and unreacted biotin was removed by dialysis. Biotinylated protein was repurified using phosphoryl-ethanolamine or PCh affinity chromatography. CRP-biotin and SAP-biotin diluted in HBS containing 3 mm EDTA and 0.005% surfactant P20 were attached to the streptavidin to 200 response units. A third empty flow cell was used as a control. Between samples of ES-62 (3-min association, 5-min dissociation, 30 μl/min) diluted in HBS (pH 7.4) containing 1 mm CaCl_2_ and 0.005% (v/v) surfactant P20, complete regeneration of flow cells was achieved with HBS containing 3 mm EDTA. Data analysis used BIAevaluation software (version 4.1.1) by separate calculation of *k_a_* and *k_d_*.

### SDS-PAGE and Ligand Blotting

ES-62 was subjected to SDS-PAGE on 12% gels, and the gels were either stained with Coomassie Blue or transferred to PVDF membranes. Molecular weight standards were Precision Plus Kaleidoscope (Bio-Rad). Membranes were blocked with 1% (w/v) BSA in PBS containing 0.05% v/v Tween 20 and 0.5 mm CaCl_2_ (PBSTC). Membranes were then incubated with 10 μg/ml CRP or TEPC15 (1 in 1000) in PBSTC or PBST containing 5 mm EDTA for 1 h at room temperature. CRP was detected with monoclonal anti-CRP (2C10) followed by anti-mouse IgG-AP conjugate (Sigma). Anti-PCh IgA binding (TEPC15) was detected with anti-mouse Ig-AP (Sigma). The chromogenic substrate was 5-bromo-4-chloro-3-indolyl phosphate/nitro blue tetrazolium (BCIP/NBT) (in sodium carbonate buffer (pH 9.6) plus 2 mm MgCl_2_). Quantitation of the relative density of protein staining in gels was performed using ImageJ.

### Analysis of Complexes

The size of complexes was determined by dynamic and static light scattering (Malvern Instruments, ζ NanoS). The samples were diluted to a final concentration of 50 μg/ml in 10 mm HEPES (pH 7.4) containing 0.15 m NaCl with no further additions or with 1 mm CaCl_2_ or 5 mm EDTA. Analysis was performed for five cycles. The standards used to confirm size were ovalbumin and thyroglobulin. HPLC was performed with a G4000SW column (300 × 10 mm) calibrated with the gel filtration markers blue dextran (V_o_), thyroglobulin, albumin, ovalbumin, and glycyl tyrosine (V_t_). Samples of ES-62 were run in a column equilibrated in HBSC buffer with or without CRP incorporated at 20 μg/ml or with EDTA to inhibit the interaction.

### Complement Assays

#### 

##### C1q Capture Assay

An adaptation of an immune complex capture assay was used to detect complexes formed between CRP and ES-62 in serum. Purified C1q (Calbiochem) was coated onto Immulon 4HB plates at 10 μg/ml overnight at 4 °C. Plates were then incubated with 3% (w/v) BSA in PBST for 2 h. Serum was diluted at 1:5; 1:10 or higher dilutions with or without added CRP in veronal-buffered saline (VBS) with 0.15 mm CaCl_2_ and 0.5 mm MgCl_2_ containing 0.2% w/v gelatin (GVBSCaMg) and ES-62 added at 1 μg/ml; 0.5 μg/ml, 0.1 μg/ml, or no ligand was added. The plate was incubated at room temperature for 1 h and then washed, and bound CRP was detected with anti-CRP. Bound protein was also analyzed by 12% SDS-PAGE and Western blotting for C1q and CRP on PVDF.

##### C1q Binding Assay

Nunc Maxisorb plates were coated with PCh-BSA, CWPS, or ES-62 at various concentrations (5.0–0.1 μg/ml) to allow equivalent binding of C-reactive protein as determined by anti-CRP as above. Plates were washed with HBSTC and blocked with the same buffer containing 1% (w/v) BSA. CRP was added at various concentrations for 1 h at room temperature, followed by various concentrations of human C1q (Calbiochem) for 1 h at room temperature and goat anti-C1q (Calbiochem). Binding was detected with anti-goat IgG alkaline phosphatase (Sigma) and 1 mg/ml *p*-nitrophenyl phosphate in bicarbonate buffer (pH 9.6) containing 2 mm MgCl_2_.

##### C3 and C4 Deposition Assays

To assess C3 deposition, ligand was coated to wells at various concentrations (ES-62, 0.25–0.05 μg/ml; CWPS, 2.5–0.5 μg/ml; PCh-BSA, 0.05–0.25 μg/ml). Human fresh serum obtained from volunteers was added to the wells at 1/100 dilution in GVBSCaMg, GVBSMgEGTA (0.5 mm MgCL_2_ and 5 mm EGTA), or GVBSEDTA (5 mm EDTA). CRP (0.4 μg/ml) or no addition was made to test or control wells. Plates were incubated at 37 °C for 30 min and washed with HBSTC. C3d deposition was detected with sheep anti-human C3d diluted 1/10,000 (Binding Site) and anti-sheep IgG alkaline phosphatase conjugate. Sera depleted of CRP were generated using adsorption with PCh-Sepharose 4B. All other sera used had endogenous CRP of less than 10 ng/ml final concentration prior to CRP addition. For iC3b, deposition plates were washed, and iC3b was detected with 1 μg/ml biotinylated antibody to the neoepitope of iC3b (Quidel), followed by streptavidin-HRP (Invitrogen). C4 deposition was measured with biotinylated polyclonal antibody to C4c, which also recognizes C4b (Dako) in combination with streptavidin HRP.

##### C2 Cleavage Assay

To determine the amount of C2a generated and bound to C4b at the plate surface, an antibody (175-62) that recognizes the active site of C3 convertase generated after C2 cleavage was used. This antibody was provided by Genentech and biotinylated using NHS-LC-biotin (Cayman Chemicals) via the protocol of the manufacturer. Plates were coated with ligand, and 50 μl of serum diluted 1/100 in GVBSMgCa, GVBSMgEDTA, or GVBSEDTA and biotinylated anti-C2a (0.7 μg/ml) with or without CRP (0.4 μg/ml) was added to replicate wells. The plates were then heated to 37 °C for 20 min, washed with PBST, and incubated with streptavidin-HRP (BIOSOURCE, 1/15,000).

##### C4-binding Protein (C4bp) Binding Assay

To assess C4 binding protein deposition, Maxisorb plates (Nunc) were coated with ligand as for the C4 binding assay and blocked with PBS containing 0.2% w/v gelatin (Sigma). Serum was diluted 1 in 100 in GVBSCaMg or GVBSMgEGTA with or without added CRP, and the plates were incubated at 37 °C for 30 min. The plates were washed, and C4 binding protein deposition was measured with biotinylated polyclonal antibody to C4bp (Serotec) diluted 1:1500. After washing, streptavidin-alkaline phosphatase (Serotec) was used at 1:3000 with *p*-nitrophenyl phosphate as substrate.

##### Factor H Binding Assay

Maxisorb plates (Nunc) were coated with ligand as above and blocked with PBS containing 0.5% w/v gelatin (Sigma). Serum was diluted 1 in 100 in GVBSCaMg or GVBSMgEGTA with or without added CRP, and the plates were incubated at 37 °C for 30 min. After washing, bound Factor H was measured using a biotinylated anti-human factor H. This reagent was generated from goat polyclonal antibody to factor H (Calbiochem) biotinylated with NHS-LC-biotin (Cayman). Detection with streptavidin-HRP was performed as described previously.

##### IgM-induced Classical Pathway Assay

Immulon 4 HBX plates were coated with 50 μl of human IgM (Sigma) at 1.6 μg/ml in 0.1 m carbonate buffer (pH 9.6) overnight at 4 °C. Wells were blocked with PBS containing 1% BSA (w/v) for 2 h at room temperature. Serum was diluted 1/100 in GVBSCaMg or GVBSMgEGTA, added to plates, and incubated for 30 min at 37 °C. IgM-activated C3d deposition was detected as described for C3 activation. To examine the effects on classical pathway activity, ES-62 was added at 0.1 μg/ml with or without CRP for 30 min at 37 °C prior to addition to the IgM-coated plates.

##### Active C4 Depletion Assay

This assay is based on the demonstration that the active thioester of C3, C4 (and α_2_-macroglobulin), can be reacted with a hydrazide and that only active protein will generate a stable labeled biotin derivative (32). This is captured by anti-C4, and the amount of derived C4 is determined using streptavidin-HRP. Serum was diluted 1/100 in GVBSCaMg or GVBSMgEGTA in the presence or absence of added CRP at 0.4 μg/ml. Individual microcentrifuge tubes containing 20 μl of diluted serum were supplemented with control buffer or ES-62 (0.5 μg/ml) or other concentration or ligand and incubated at 37 °C for 1 h. 2 m NaCl (20 μl) was added to each tube together with 2 μl of 10 mm biotin-PEG_4_-hydrazide (Thermo Scientific) in DMSO. The tubes were then heated to 52 °C for 1 h and cooled. The reaction mixture was left overnight in the fridge before the assay.

Nunc Maxisorb plates were coated with affinity-purified goat anti-C4 (0.2 μg/ml, Immune Consultant Laboratories), and plates were blocked with PBST containing 2% (w/v) BSA. Samples treated as above were diluted to the point at which they gave a linear response in the assay (1–800) and incubated at room temperature for 2 h. A standard curve on each plate was generated from a treated C4 standard. Bound biotin-labeled C4 was determined using streptavidin-HRP conjugate (BIOSOURCE).

### Synthesis of AGP-PCh

AGP was purified from human serum using anion exchange chromatography (DE52), Cibacron-Blue-Sepharose, and then another anion exchange stage to purify AGP1 (modified from Ref. 33). It was dialyzed into 0.1 m phosphate buffer (pH 7.4) containing 0.15 m NaCl and then oxidized to target mainly sialic acid with 2.5 mm sodium periodate for 1 h at room temperature. The protein was desalted on a PD-10 column (GE Healthcare) into 0.1 m phosphate buffer, and *p*-aminophenyl-PCh was added at 1 mm final concentration (∼50× molar excess), followed by sodium cyanoborohydride to give a final concentration of 50 mm. After incubation in the dark overnight at 4 °C, the protein was again desalted on a PD-10 column.

### Deglycosylation of AGP-PCh and ES-62

PNGase F (Promega) was used to remove the *N*-linked carbohydrate according to the protocol of the manufacturer. 12.5 μg of AGP-PCh or 10 μg of ES-62 was denatured and digested with 2 units of PNGase in phosphate buffer for 3 h at 37 °C. The removal of PCh was confirmed by Western blotting using the anti-PCh myeloma protein TEPC15 and anti-mouse immunoglobulin-alkaline phosphatase and detection with 5-bromo-4-chloro-3-indolyl phosphate/nitro blue tetrazolium substrate.

### Synthesis of BSA-PEG_4_-PCh

10 mm propargyl-NHS (Sigma) in DMSO (600 μl) was incubated with 10 mg/ml BSA (3 ml) in PBS at room temperature for 2 h. The BSA-alkyne generated was desalted on a PD10 column.

2 mg *p*-aminophenyl-phosphorylcholine was dissolved in 1.0 ml 0.1 m MES (pH 4.5) and added to an equal volume of 1 mg/ml azide-PEG_4_-COOH (Iris Biotech) and 0.2 ml 10 mg/ml 1-ethyl-3-(3-dimethylaminopropyl) carbodiimide and incubated overnight at room temperature. The azide-PEG_4_-phenyl-PCh was separated from the smaller precursors on a Biogel P2 column (15 ml, Bio-Rad) washed with PBS. Click chemistry between azide and alkyne was used to generate the BSA-PEG_4_-PCh conjugate. The reaction was composed of 1.0 ml of 0.2 mm BSA-alkyne and 0.7 ml of 0.3 mm azido-PEG_4_-phenyl-PCh, 2 μl of 500 mm fresh ascorbic acid, 50 μl of copper/Tris-[(1-benzyl-1H-1,2,3-triazol-4-yl) methyl]amine (TBTA) reagent (1 volume of 10 mm copper II/2 volumes of 50 mm TBTA, Sigma), and the reagents were incubated for 2 h with rolling. The BSA-PEG_4_-PCh-containing fractions were collected after desalting on a PD10 column.

## Results

### 

#### 

##### CRP Binds to ES-62 with High Avidity

**FIGURE 1.**
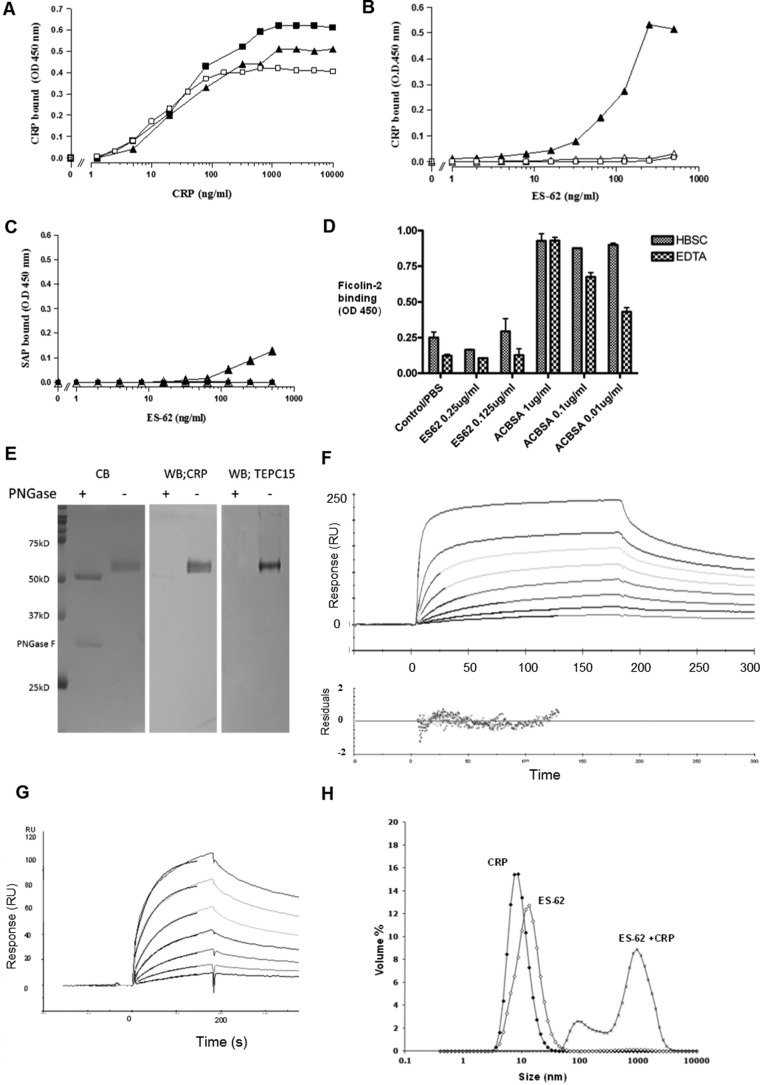
**High-avidity binding of C-reactive protein to ES-62 is calcium-dependent and can be inhibited by PCh.** *A*, dose response of binding of purified CRP to immobilized ES-62 (2.0 μg/ml, ■), PCh-BSA (0.5 μg/ml, ▴), or CWPS (5 μg/ml, □) on microtiter plates. Various concentrations of CRP were offered, and binding of CRP was detected using polyclonal anti-human CRP-HRP. *OD*, optical density. *B*, CRP binding from ES-62 is calcium-dependent and can be inhibited by PCh. Various concentrations of ES-62 were coated onto microtiter plates, and normal serum diluted 1 in 50 to give a final CRP concentration of 50 ng/ml was added. Binding of CRP was detected with the anti-native human CRP monoclonal antibody 2C10 and anti-mouse IgG HRP and 1,1,3,3 tetramethylbenzidine substrate (optical density, 450 nm). Serum was diluted in HBS containing 1 mm CaCl_2_ (▴), HBS with 10 mm EDTA (▵), or HBS with 1 mm CaCl_2_ and 50 mm phosphorylcholine (□). *C*, SAP provided in serum diluted 1 in 50 binds weakly to ES-62 (▴) but not PCh-BSA-coated plates (■). SAP was determined using monoclonal anti-SAP and anti-mouse IgG HRP. Controls show binding to ES-62 in the presence of EDTA (▵). *D*, plates were coated with ES-62 or the positive control acetylated BSA (*ACBSA*) at various concentrations, serum was added in the presence or absence of calcium, and binding was detected with biotinylated anti-ficolin 2 and streptavidin HRP. Data are mean ± S.E. of triplicates. *E*, ligand blotting of ES-62 following SDS-PAGE demonstrates binding of C-reactive protein to PCh attached to *N*-linked glycan. *Left panel*, ES-62 or ES-62 deglycosylated with PNGase stained directly with Coomassie Blue (*CB*). *Center panel*, ES-62 was transferred to PVDF, and CRP binding in TBSC was detected with anti-CRP and anti-mouse-alkaline phosphatase. *Right panel*, as for the *center panel*, but PCh was detected with anti-PCh myeloma protein, TEPC15. *WB*, Western blotting. *F*, surface plasmon resonance analysis of interaction. ES-62 was immobilized, and CRP was offered at concentrations of 10, 2.5, 1.25, 0.62, 0.3, 0.16, 0.08, and 0.04 μg/ml. Langmuir 1:1 analysis was performed. Residuals from the association analysis are shown below. *RU*, response unit. *G*, surface plasmon resonance analysis of ES-62 (12.5, 6.25, 3, 1.6, 0.8, 0.4, and 0.2 μg/ml) binding to biotinylated CRP immobilized on a streptavidin surface. The *superimposed lines* show modeled fit. *H*, CRP and ES-62 form large complexes in fluid phase. The size of the complex was determined using light scattering 5 min after mixing for 50 μg/ml CRP and 65 μg/ml ES-62 in HBS in the presence of 1 mm CaCl_2_.

We immobilized ES-62 on a microtiter plate and examined its ability to bind purified CRP in comparison with the known natural ligand CWPS and a synthetic ligand, PCh-BSA. CRP showed concentration-dependent binding to ES-62 with a similar concentration required for half-maximal binding to each of the three ligands (Fig. 1*A*). Coating the plate with concentrations of ES-62 as low as 10–20 ng/ml, we were able to detect binding of CRP from serum (Fig. 1*B*). This binding of CRP was detectable with a monoclonal antibody to native CRP and could be inhibited with EDTA or PCh, thereby confirming that the binding was calcium-dependent and through the PCh-binding site of CRP. Although no SAP could be seen binding to PCh-BSA, the interaction of SAP could only be observed at the highest concentrations of immobilized ES-62 (Fig. 1*C*). Little or no IgM binding was detected, probably because IgM specific for PCh is reported at low concentrations in human serum. Despite the reported high amount of terminal *N*-acetylglucosamine in ES-62 (5), ficolin-2 was not bound (Fig. 1*D*). ES-62 was then subjected to SDS-PAGE, and, following transfer to a membrane, we used ligand blotting with CRP and anti-CRP to demonstrate that the CRP bound directly to the ES-62 (Fig. 1*E*). CRP binding required the presence of the PCh on the *N*-linked carbohydrate because PNGase F digestion abrogated CRP (and anti-PCh) binding (Fig. 1*E*)). Initial plate experiments (Fig. 1, *A–C*) suggested a high avidity of binding. To confirm this, we used surface plasmon resonance with immobilized ES-62 and demonstrated a major high avidity component with a *K_d_* of ∼1 × 10^−10^ m, although complex binding kinetics are to be expected in pentameric and multimeric ligand interactions because some analyte will bind at more than one site (Fig. 1*F*). Off-rates varied between 0.6–1.4 × 10^−3^ s^−1^ at different concentrations, but on-rates were very fast: 3 × 10^6^-10^7^ m^−1^s^−1^. In the reverse orientation (ligand and analyte exchanged with each other), we employed a gentle way to label CRP with biotin at neutral pH and captured this on a surface of immobilized streptavidin. The off-rate (*K_d_*) was highly reproducible in the range of 1.4–1.3 × 10^−3^ s^−1^ over the range of concentrations tested, but the on-rate fit was less consistent, between 1 × 10^5^ and 1 × 10^6^ m^−1^s^−1^. The *K_d_* in this orientation was slightly lower, between 0.8 and 3 × 10^−9^ (Fig. 1*G*). SAP immobilized in this orientation failed to bind to ES-62.

##### Purified ES-62 and CRP Can Form Large Complexes in Fluid Phase

It was of interest to discover whether the interaction between CRP and ES-62 was capable of generating complexes in fluid phase. In preliminary experiments, we analyzed ES-62 alone in buffer containing 1 mm CaCl_2_ using a gel filtration column that eluted at the appropriate molecular weight (∼240 kD), running as a tetramer as reported previously. When CRP was incorporated into the buffer of this gel filtration system and ES-62 was injected, no peak of ES-62 was observed. Rather, a depletion of CRP was observed, consistent with formation of a complex too large to enter the column. When the same experiment was repeated in the presence of EDTA or in calcium without CRP, ES-62 was observed at its expected molecular weight. To confirm the interaction, we used non-invasive light backscattering to show that adding ES-62 with an equimolar concentration of CRP led to all CRP and ES-62 forming a large complex 0.1–2 μm in diameter (Fig. 1*H*).

##### CRP Is the Major ES-62-binding Protein in Serum

**FIGURE 2.**
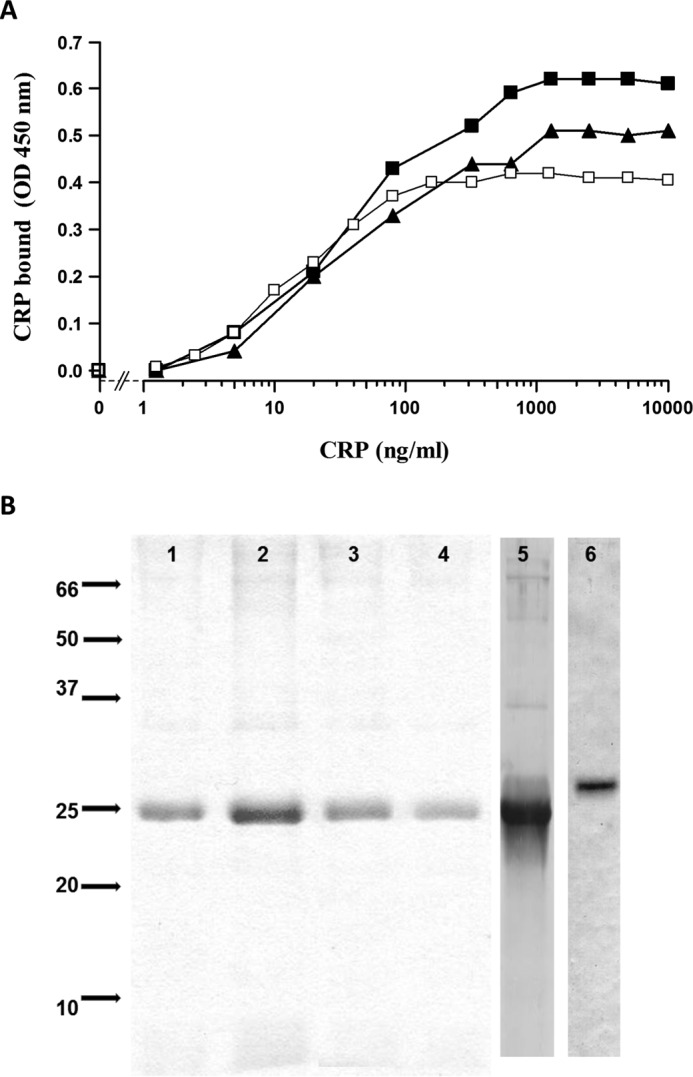
**CRP is the major serum protein binding to ES-62.** *A*, other serum proteins do not inhibit CRP binding to ES-62. ES-62 (0.25 μg/ml) was coated onto microtiter plates, and CRP at indicated concentrations was added alone (□) or in the presence of two individual normal serum samples (■ and ▴). CRP binding was detected as for Fig. 1*B. OD*, optical density. *B*, CRP is the major protein pulled out of normal serum by ES-62 (*lanes 1* and *2*) or AGP-PCh (*lanes 3* and *4*) coupled to magnetic beads. Bound protein was eluted with EDTA (*lanes 1* and *3*) or by SDS-PAGE lysis buffer (*lanes 2* and *4*). SDS-PAGE gels were run and analyzed by Coomassie Blue staining (*lanes 1–4*). Gels were also immunoblotted, and CRP eluted from ES-62-coated beads was detected by Western blotting (*lane 5*), as was SAP to a lesser degree (*lane 6*).

CRP binding to ES-62 was similar whether or not serum was present, suggesting that there was little or no competing protein in serum and, therefore, CRP was the major ES-62-binding protein (Fig. 2*A*). To further confirm this, we initially used ES-62-Sepharose 4B to examine the binding of serum proteins and detected the major specific bands eluted as CRP and SAP (data not shown). SAP is known to bind minor components of agarose, and given potential for cross-reaction with the support, we then coupled ES-62 to an amine-coated magnetic silica bead support and re-examined proteins pulled out of serum. This confirmed that CRP was the major protein bound from serum (Fig. 2*B*), comprising 80–85% of eluted protein by gel densitometry, whether this was calcium-dependent (EDTA elution) or not (SDS lysis buffer elution). On this support, only trace amounts of SAP were bound.

##### CRP Bound to ES-62 Does Not Generate C3 Degradation Products

**FIGURE 3.**
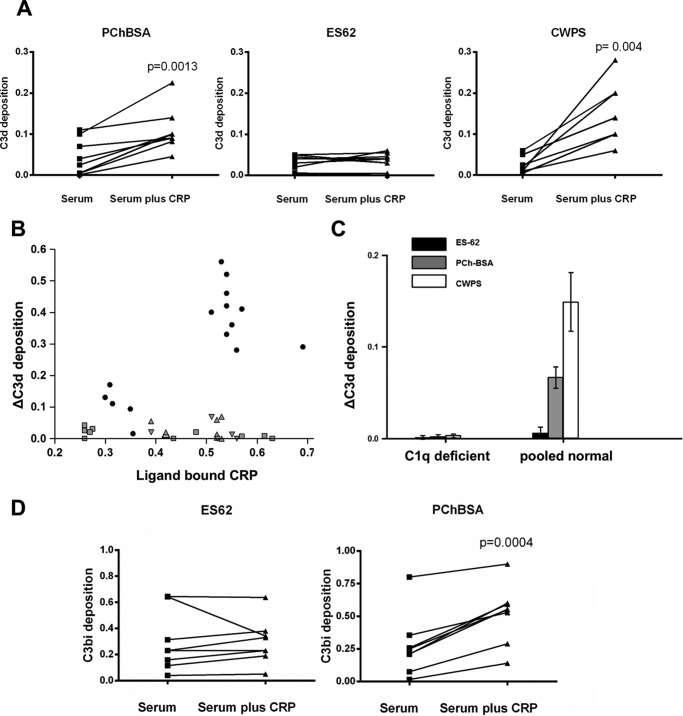
**ES-62, in contrast to PChBSA and CWPS, does not lead to C3d or C3bi deposition.** *A*, CRP increases deposition of C3d onto PCh in PChBSA and CWPS but not ES-62. Individual sera from healthy donors with or without added CRP in VBSCaMg were incubated at 37 °C in ligand-coated plates in VBSCaMg, and C3d deposition was determined. The background level of complement activation for ES-62 seen without added CRP was not diminished in sera depleted of PCh binding activity by passage through an anti-PCh-Sepharose column. Statistical analysis was undertaken by paired *t* test. *B*, the same experiment was performed, but the increase in C3d deposition mediated by CRP was plotted against the amount of CRP bound to the plate under each condition. Ligands: PCh-BSA (■), CPWS (●), or ES-62 (▴). *Small symbols* represent data obtained for individual donor serum. *Larger symbols* represent data for pooled serum. *C*, complement activation by ES-62 and other PCh ligands is through C1q. Normal serum or C1q-depleted pooled sera were used to determine C3d deposition against ES-62, PCh-BSA, and CWPS. Data are mean ± S.E. of four replicates. *D*, CRP addition to serum increases complement C3bi deposition to ligand PCh-BSA but not ES-62. Following incubation as in *A* at 37 °C for 30 min, C3bi bound to the surface was detected with biotinylated anti-C3bi and streptavidin HRP. In *A*, *B*, and *D*, the data are for between seven and nine different donors measured in three different experiments.

The PCh ligand of CRP in ES-62 is present on the end of a long and flexible carbohydrate chain, and it was uncertain how this might affect the ability of CRP to activate complement. Pooled or individual normal donor sera with a CRP concentration of less than 1 μg/ml were diluted to give a final CRP of less than 10 ng/ml, and native CRP was added at 400 ng/ml. Complement activation was assessed using ligand-coating concentrations chosen to lead to equivalent CRP bound. CRP increased C3d deposition onto the PCh-containing ligands PCh-BSA and CWPS, but no increase was seen with ES-62 (Fig. 3*A*). We assayed the amount of CRP bound in these interactions, and to rule out effects because of different CRP binding, we repeated the experiment with a range of CRP concentrations. However, even at the higher amounts of CRP bound to ES-62, little or no additional C3d was deposited in comparison with the other ligands (Fig. 3*B*). Because CRP has been reported to activate complement through factor H-like proteins (32) and lectin pathway activation may be a contributor to C3 deposition, we determined whether the activation was C1q-dependent using C1q-deficient sera. Activation was reduced to background in the absence of C1q (Fig. 3*C*), showing that CRP worked exclusively through C1q and that the classical/CRP-mediated pathway was predominant in CRP-mediated increases in C3d deposition. We also checked for formation of another C3 cleavage product, C3bi, which again was induced by PCh-BSA but not ES-62 (Fig. 3*D*).

##### CRP Bound to ES-62 Recruits C1 and Cleaves C4

**FIGURE 4.**
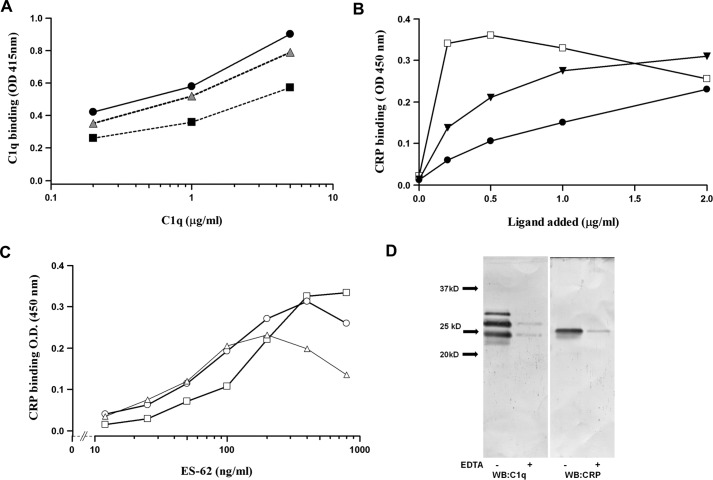
**Demonstration of the CRP·ES-62·C1q complex in serum.** *A*, C1q binds to CRP/ES-62. ES-62 (▴), PCh-BSA (■), or *S. pneumoniae* CWPS (●) were immobilized on microtiter plates at concentrations that lead to equivalent CRP binding, determined by polyclonal anti-CRP binding. CRP (0.4 μg/ml) was added, followed by C1q at various concentrations (0, 0.2, 2.0, and 5.0 μg/ml), and following washing, bound C1q was detected with anti-C1q-AP and *p*-nitrophenyl phosphate substrate (optical density (*OD*) 415). *B*, CRP binds to C1q only when ligand is added to serum. Plates were coated with C1q, and ligand (□, CWPS; ▾, PCh-BSA; ●, ES-62) was added to wells, with serum diluted 1 in 5 in VBSCaMg so that the final CRP concentration was 0.4 μg/ml. Complex was measured by CRP binding, determined using polyclonal anti-CRP -HRP. *C*, maximal CRP: ES-62 complex is captured to C1q at equal molarity of CRP and ES-62. Plates were coated with C1q, and serum was diluted at 1:100 added with CRP at 0.03 μg/ml (▵), 0.06 μg/ml (○), and 0.125 μg/ml (□). Various amounts of ES-62 were added, and CRP bound to the C1q was measured using an anti-CRP monoclonal antibody as in Fig. 1*B. D*, C1q was recruited to CRP bound to ES-62. Plates were coated with ES-62 and incubated with serum diluted in VBSCaMg or VBSEDTA at 4 °C. The plates were washed, and bound protein was removed from the surface with SDS sample buffer, run on a 12% SDS-PAGE gel, and Western-blotted with anti-C1q and CRP. *Lanes 1* and *2*, blotting for C1q (to reveal C1qA, C1qB, and C1qC chains); *lanes 3* and *4*, blotting for CRP. Representative data from two to three donor sera are shown.

We wanted to determine at what stage CRP bound to ES-62 failed to activate complement. Therefore, we tested the ability of purified C1q to bind to CRP when bound to CWPS, PCh-BSA, or ES-62. Immobilized ES-62 with bound CRP was as efficient at binding purified C1q as other ligands (Fig. 4*A*). To determine whether the interaction took place in whole sera, plates were coated with C1q and ES-62 or other ligand added to sera, and CRP-ligand complex binding was determined. C1q did not capture CRP in the absence of ligand, but when any of the three ligands was added to the sera, then CRP could be detected binding to C1q (Fig. 4*B*). The maximal binding was observed at an approximate equivalence of molar amounts of CRP and ES-62 (Fig. 4*C*). Such plates were washed, and bound material was analyzed by Western blotting, and the binding of CRP and C1q was shown to be calcium-dependent (Fig. 4*D*).

**FIGURE 5.**
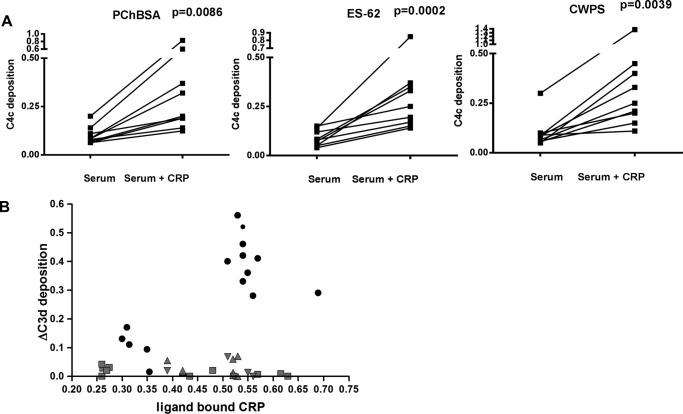
**ES-62-bound CRP leads to C4 deposition.** *A*, CRP bound to ES-62 leads to C4 product deposition. Plates were coated with ES-62 or other CRP ligand at concentrations that bound similar amounts of CRP. Serum diluted in VBSCaMg from eight to ten normal healthy donors was added with or without CRP at 0.4 μg/ml and incubated at 37 °C for 30 min. Deposited C4c was determined with biotinylated anti-C4c and streptavidin HRP. Statistical analysis was undertaken by paired *t* test (*n* = 9). *B*, bound CRP correlates with increased C4c deposition for all three ligands. Data were obtained as in *A*, but different serum and CRP concentrations were used, and bound CRP was measured and plotted against the deposited C4c. PChBSA, ■; ES-62, ●; CWPS, ▴.

We then employed an assay of C4 deposition, which was similar to that for C3d deposition. ES-62 and the other positive control ligands all resulted in CRP complexed with C1 and cleaved C4 (Fig. 5*A*). There was a correlation between the amount of bound CRP and the deposition of C4 (Fig. 5*B*; linear correlation was significant at p = 0.02, 0.01, and 0.03 for CWPS, ES-62, and PCh-BSA, respectively).

##### Failure of the ES-62·CRP Complex to Lead to Activated C3 Convertase Does Not Lie with Recruitment of C4-binding Protein or Factor H

**FIGURE 6.**
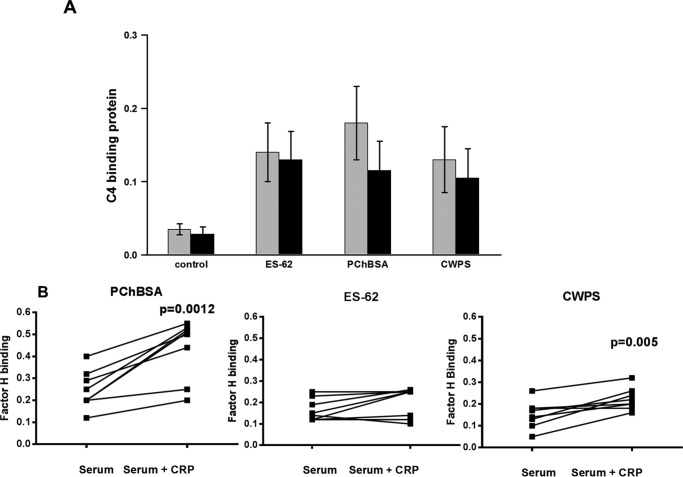
**Lack of a role for complement regulatory factors.** *A*, no significant difference between binding of C4 binding protein to immobilized ES-62-, PChBSA-, or CWPS-coated microtiter plates. Plates were coated with ES-62, PCh-BSA, or CWPS as described previously and incubated with serum with (*black bars*) or without CRP (*gray bars*). Following incubation for 30 min with serum diluted in VBSCaMg or VBSMgEGTA buffer with or without CRP (0.4 μg/ml), the plates were washed, and C4bp was detected with biotinylated antiC4bp. Data are presented as mean ± S.E. of five different donor sera. *B*, factor H recruitment to the plate surface following CRP-mediated complement activation in response to PCh-BSA and CWPS but not ES-62. PCh ligand was coated to the plate to recruit equivalent CRP amounts, and following incubation with serum with or without added 0.4 μg/ml CRP at 37 °C for 30 min, the amount of factor H bound was determined. Two experiments on eight different donors were analyzed by paired *t* test.

Complement control proteins have been reported to interact with CRP. However, no increase in C4bp recruitment to ES-62-CRP was observed. C4bp was not involved in the failure of ES-62·CRP·C1q to result in an activated C3 convertase (Fig. 6*A*). The amount of factor H recruitment to PCh-BSA- and CWPS-coated plates during incubation was increased by the presence of CRP in serum, but this was not seen with ES-62 (Fig. 6*B*). This was consistent with the idea that C3 convertase was not generated downstream of CRP binding to ES-62.

##### Failure of the ES-62·CRP Complex to Result in Activated C3 Convertase Lies with the Inability to Cleave C2

**FIGURE 7.**
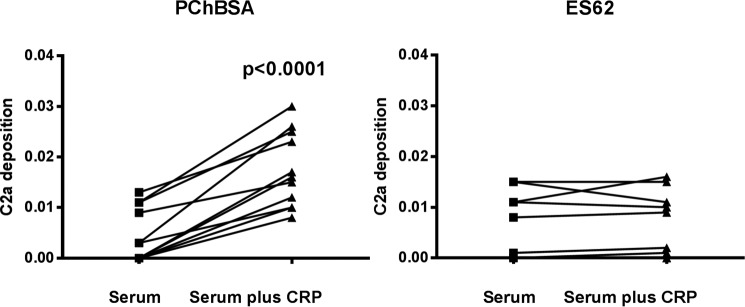
**ES-62 does not efficiently generate a C3 convertase.** CRP addition to serum leads to active C2a generated in response to immobilized PCh-BSA but poorly in response to immobilized ES-62. Plates were coated with ligand as described previously and incubated at 37 °C for 30 min, and active C2a was detected with the biotinylated monoclonal antibody 175-62. Statistical analysis was undertaken by paired *t* test on 11 different sera in two different experiments.

The mechanism of C3 convertase formation requires that C2 binds to C4b generated close to the C1 complex and that the C1s cleaves the C2 before the C4b is inactivated. Using a complement activation assay based on a monoclonal antibody that recognizes only active C2a, we observed an increase in C2a formation mediated downstream of CRP binding to PCh-BSA but not ES-62 (Fig. 7). Therefore, although equivalent C4b is generated in response to the ES-62·CRP·C1 complex, the efficiency of cleavage of recruited C2 is greatly reduced.

##### High Flexibility of Phosphorylcholine-containing Ligands Leads to Failure of Bound CRP to Activate Complement

**FIGURE 8.**
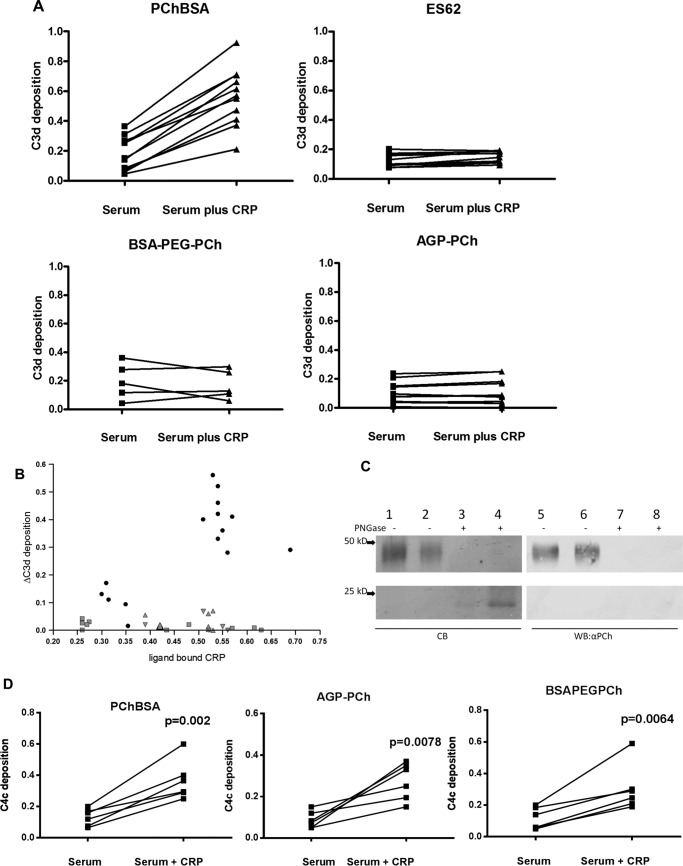
**Highly mobile PCh ligands bind CRP but do not activate complement.** *A*, CRP increases deposition of C3d onto PCh in PChBSA but not ES-62 or the synthetic PCh ligands AGP-PCh and BSA-PEG4-PCh. Individual sera from healthy donors with or without added CRP in VBSCaMg were incubated at 37 °C in ligand-coated plates in VBSCaMg, and C3d deposition was determined. Statistical analysis was undertaken by paired *t* test on 5–11 different sera in two different experiments. *B*, the same experiment was performed, but the increase in C3d deposition mediated by CRP was plotted against the amount of CRP bound to the plate under each condition when ligand concentration was varied. Ligands: PCh-BSA (●), ES-62 (▴), AGP-PCh (▾), and BSA PEG_4_ PCh (■). *C*, PCh of synthetic PCh-AGP is attached to *N*-linked carbohydrate. Shown is SDS-PAGE and Coomassie Blue (*CB*) staining (*lanes 1–4*) or immunoblot (*lanes 5–8*) with anti-PCh (TEPC15) of AGP-PCh and an equivalent amount of PNGase-treated AGP-PCh. *Lanes 1*, *4*, *5*, and *8*, 5 μg/ml; *lanes 2*, *3*, *6*, and *7*, 2.5 μg/ml. *WB*, Western blotting. *D*, CRP addition to serum increases C4c deposition onto AGP-PCh and BSA PEG_4_-PCh. Data for six different donor sera were analyzed by paired *t* test.

This failure to induce C3 convertase formation was related to the lack of C2 cleavage despite equivalent C4 activation. We postulated that this was due to the flexibility of the ligand for CRP and, consequently, the bound CRP and bound complex. The C1 complex with active C1s would cleave the C4 that would bind to a reactive site near the complex, but when C2 subsequently interacts with the C4, the enzyme is no longer close enough to efficiently cleave the C2 to generate C4bC2a (C3 convertase). To investigate this, we considered that a PCh ligand placed on any similarly sized glycan chain would share this property. To generate an analogue, we used α_1_-acid glycoprotein (AGP), which has four *N*-glycans largely terminating in sialic acid, and added PCh through reductive amination. This ligand also pulled out only CRP from human serum (Fig. 2*B*). For analysis of complement activation, the PCh ligand density was adjusted to give similar levels of CRP binding to the plate. This synthetic version of the glycan with terminal PCh also failed to activate C3, as shown by no additional C3d deposition on CRP addition (Fig. 8*A*). The PCh on this AGP was carbohydrate-linked because PCh was not detected after treatment with PNGase F, as shown by SDS-PAGE and Western blotting (Fig. 8*C*).

Because this ligand is also carbohydrate-bound, we generated a further synthetic version with a highly flexible PCh using a PEG spacer that had an approximately similar length as ES-62 carbohydrate (35). This was generated by initial carbodiimide coupling of *p*-aminophenyl PCh and azide-PEG_4_-COOH to produce azide-PEG4-phenyl-PCh and then click chemistry to couple this to an alkyne derivative of BSA. CRP bound to this ligand generated no additional C3d deposition. This was in contrast to when comparable amounts of CRP were bound to BSA-PCh ligand lacking the spacer but that was otherwise identical, thus indicating again that the feature of the PCh that was important was its presence on a terminal flexible spacer (Fig. 8*B*). The comparable binding of CRP to these ligands was also reflected in the analogous cleavage and deposition of C4 following CRP binding to AGP-PCh and BSA-PEG-PCh (Fig. 8*D*), as demonstrated previously for ES-62.

##### The ES-62·CRP Complex Depletes Classical Pathway Activation and the Rate-limiting Component C4

**FIGURE 9.**
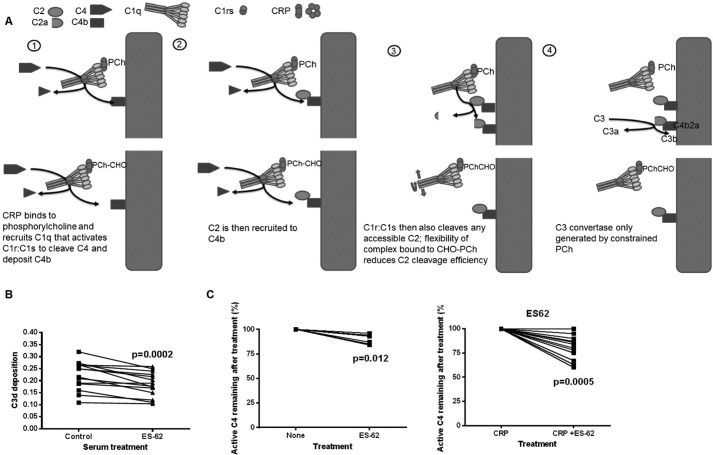
**ES-62 added to serum reduces classical complement activation and the rate-limiting factor active C4.** *A*, diagram of the effects of different PCh forms on the complement pathway. *B*, classical complement activation was measured by C3d deposition onto IgM-coated microtiter plates. Serum diluted 1 to 100 in VBSCaMg was either left untreated or treated with ES-62 (0.1 μg/ml) for 30 min at 37 °C prior to addition to the IgM. Statistical analysis was undertaken by Wilcoxon matched pairs test (*n* = 14 different sera in three different experiments). *C*, active C4 was measured following addition of ES-62 (0.5 μg/ml) to serum diluted 1 in 100 and incubation for 45 min at 37 °C (*n* = 6 different donor sera). *D*, as for B, but CRP was added at 0.4 μg/ml final concentration. Data for 12 different donor sera in two separate experiments were analyzed by Wilcoxon matched pairs test.

Flexible ligand had initiated C4 cleavage, as summarized in Fig. 9*A*, but failed to activate C3 convertase. To assess whether ligand such as ES-62 present in serum during infection and CRP would alter complement activity, we added the nematode product to serum and then examined the ability of that serum to be activated by an IgM-driven classical complement pathway assay. We measured C3d deposition following activation with surface-immobilized IgM. When ES-62 was added at a concentration of 0.1 μg/ml (enough to complex all available CRP in the diluted serum), there was a consistent and statistically significant reduction in the capacity of the sera to deposit C3d (Fig. 9*B*). It has been determined previously that the concentrations of C2 and C4 are rate-limiting for classical pathway activation. The likely explanation for this effect was that the C4 was depleted (36). Therefore, we added ES-62 to the sera and incubated at 37 °C to determine whether significant C4 was depleted. The assay for C4 relied on biotinylation of the thioester in only active C4 remaining after incubation. Indeed, the amounts of active C4 were depleted by ES-62, and the amounts of C4 depleted (∼5–35%) were approximately equivalent to the reduction seen in the classical pathway activation assay (Fig. 9*C*). Predictably, addition of CRP to a concentration comparable with ES-62 lead to a greater depletion (Fig. 9*D*).

## Discussion

ES-62 derived from the rodent filarial nematode *A. viteae* represents a readily available model protein for studying the role of PCh on proteins derived from filarial nematode species that parasitize humans. This is particularly the case because PC-containing glycans, including chito-oligomers, and an identical PCh-glycan structure to that found on ES-62 have been found in the human filarial nematode parasite *O. volvulus* (5).

The data in this report show that ES-62 binds strongly to CRP in a PCh-dependent manner. The interaction of phosphodiester-linked PCh has been shown to be about 10-fold weaker than monoester PCh (14). However, the strong binding of ES-62 for CRP reinforces previous data that suggest that a phosphodiester can be a strong ligand when linked to carbohydrate (37). The binding of CRP to ES-62 is complex, as expected for two multimeric proteins, and multiple different protein complexes may be formed. However, a significant component has a high avidity, as shown by surface plasmon resonance, which gave a similar avidity in either orientation. Large complexes are formed when purified proteins are mixed at equivalent concentrations in the presence of calcium. Complexes were also formed in serum, as shown by the capture assays using C1q, which demonstrated maximal binding at approximately equivalent CRP-ES-62 concentrations. These assays also demonstrate an important point: that CRP and C1q do not interact unless the CRP has bound ligand, implying that a conformational change is required in CRP to allow this binding. Models of the binding of CRP to C1q imply that CRP may need to undergo some conformation change, suggesting that the interaction is a strained one (20). In addition, the major protein in human serum interacting with ES-62 was CRP. No binding of ficolin 2 was seen, despite the observations that it bound PCh attached to teichoic acid (38). It is possible that binding to GlcNAc could be prevented by PCh substitution. We could see little binding of IgM or IgG from serum of normal healthy donors, but this may change in individuals with an induced anti-PCh antibody response.

Our demonstration that, despite interacting with CRP, ES-62 did not activate C3 was unexpected. We confirmed that there was no difference between C4bp recruitment to C4b with the different PCh-containing ligands under study. There have been reports that high concentrations of CRP can interact with factor H (27), but here the concentrations were in the normal range, and we saw no increase in factor H recruitment except when associated with complement activation. In our study, all CRP activity was entirely dependent on C1q, thus other reported CRP activities, for instance through factor H-related 4 (34), were not involved. Therefore, ES-62 was capable of binding CRP and causing the change in CRP that is needed for binding of C1q, in turn leading to the rearrangement of C1q that drives reorientation of its globular headgroup and the proposed activation of C1r and, subsequently, C1s (39). That a flexible ligand can do this also provides more evidence for a strained to relaxed driving force for C1r/C1s activation (40).

The following question then arises: because we can observe a strong activation and deposition of C4, why is there no C3 cleavage and thus no C3 convertase generated? PCh on ES-62 is present on a highly flexible *N*-linked glycan. When the C4 is cleaved and C4b binds to a region of ES-62 or CRP or a neighboring attachment site, then the CRP-activated C1 complex is still highly mobile and would likely move before it can cleave C2. Variation in efficiency of generating C3 convertase has been observed before; for instance, lectin pathway activation is much more efficient than the classical pathway (41, 42). The reason for this is the relative off-rate of cleaved C4b. From mannose-binding lectin (MBL)/MASP-2, the off-rate of C4b is relatively slow, and because of the short half-life of the thioester of C4b, this means that, when it attaches to a surface, it is closer than for C4b generated by C1s with its faster off-rate, which can then attach further from the C1s, which then makes C2 cleavage less favorable. Normally, for the classical pathway, it requires four C4b molecules to generate one convertase (43). The ES-62·CRP·C1 complex containing the active C1s generates C4b, which will attach to a local site, but the highly mobile nature of the ligand will lead to movement of the C1s away from the site where C4b attached, preventing interaction with C2 when it binds to C4b. In this way the efficiency of generation of C3 convertase becomes very low.

This hypothesis was confirmed when we generated PCh ligands that had different protein components and different flexible components where the only similarity was the mobility and the PCh. These behaved in the same way as ES-62 in terms of complement activation.

An important conclusion from this work is that it emphasizes the importance of the three-component stage when C4b recruits C2, and then the C1 complex needs to cleave that C2 to generate the C3 convertase as the rate-limiting step in the classical pathway. In devising the assay of C3 convertase generation using the monoclonal antibody to C2a, we were able to monitor this stage of complement activation when alternative assays involving Western blotting assays of C2 products were at best hard to quantitate. This methodology will prove useful in the examination of the efficiency of this stage of complement activation.

ES-62 is most likely to be found in solution *in vivo*, and this would also restrict the chances of generating a C3 convertase by the demonstrated depletion in C4 concentration. C4 is rate-limiting, and not only does depletion reduce classical pathway activation, but addition of C4 has been shown to increase it (36). This may have relevance in evasion of the immune response by filarial nematodes because it is known that these organisms are capable of activating the complement system (44), and hence they may have evolved strategies to try and minimize this. It has been observed, for example, that microfilaria larva stages of the human parasite *Loa loa* acquire regulatory proteins from the host to evade complement attack (45). The observed effects of ES-62 could also help reduce unwanted pathology. In lymphatic filarial infection, PCh-containing molecules have been shown to be particularly detectable in patients with circulating microfilariae (larval forms) but without overt disease, in most cases at a serum concentration of about 0.1–1.0 μg/ml (12). This concentration is similar to that of CRP in the normal serum range and thus is able to make complex efficiently. It has also been demonstrated in another study that this patient group has levels of CRP that are not much above normal levels, whereas, in people with overt pathology, CRP levels are greatly elevated (46). The latter study also showed an inverse correlation between serum levels of CRP and PCh-containing molecules. This raises the possibility that a contributing factor to the induction of pathology may be a low level of PCh-containing molecules, resulting in a lack of early complement component depletion via CRP.

The presence of PCh has been demonstrated in many non-filarial helminth parasites (17), including *Ascaris suum* (47), *Hymenolepis diminuta* (48), *Toxocara canis* (49, 50), and *Echinococcus granulosus* (51), and in some cases it has been found to be associated with glycans and/or to interact with CRP. In addition, the protozoan *Leishmania donovani* promastigotes express flexible carbohydrate ligands (repeating phosphodiesters) for CRP (37, 52). However, whether the immune evasion mechanism we describe for ES-62 in this manuscript applies to these other organisms remains to be established. In addition, some parasites, including strains of *Trichomonas vaginalis*, express *N*-linked phosphorylethanolamine modifications (53), and this raises the question whether SAP leads to a similar incomplete complement activation.

Finally, also of note, complement has always been regarded as a vital component of the innate response, but more recently it has gained importance as a regulator of humoral and cellular immunity through immune complex and/or C1q or ligation of complement receptors (27, 54). The balance between effects on T cells and antigen-presenting cells through activation products of complement such as C3a or complexes of C1q is considered an important determination of downstream immune responses in antimicrobial activity and also autoimmunity (46). The evasion strategy we describe is likely one of many that help nematode survival through contributing to reduced complement activation and possibly associated consequences on adaptive immunity. At the same time, it is interesting to speculate that the loss of such parasite manipulation of the host could also contribute to the increase in chronic inflammatory conditions, and this is often considered in relation to the “old friends or hygiene hypothesis.” It is thus pertinent to consider the therapeutic potential of a molecule with properties such as ES-62 in these diseases. Certainly, given the leading role of CRP in the general inflammatory response, a PCh-containing molecule, which interferes with its activity, could have widespread clinical application but particularly in medical emergencies such as myocardial infarction and stroke, where prompt control of the inflammatory storm is mandatory. The use of a bisphosphocholine small molecule that complexes CRP was shown to reduce inflammation and complement mediated damage by depleting CRP (55). Future studies should thus now extend to assessment of clearance of CRP and *in vivo* effects in models of inflammatory disease. In addition, designing drug-like analogues with similar effects to ES-62 may be a prudent path to take.

## Author Contributions

U. K. A., N. C. M., A. J. I., L. A. R., and J. G. R. performed the experiments. All authors contributed to the manuscript, and J. G. R. and W. H. were responsible for the overall project design and concept.

## Footnotes

4 The abbreviations used are:

PCh: phosphorylcholine
CRP: C-reactive protein
CWPS: cell wall polysaccharide
SAP: serum amyloid P component
AGP: α_1_-acid glycoprotein
HBS: HEPES-buffered saline
VBS: veronal-buffered saline
TBTA: Tris-[(1-benzyl-1H-1,2,3-triazol-4-yl) methyl]amine
AP: alkaline phosphatase.
